# DFENet: Deep Feature Enhancement Network for Accurate Calculation of Instantaneous Wave-Free Ratio

**DOI:** 10.1109/JTEHM.2020.2999725

**Published:** 2020-06-03

**Authors:** Jiping Li, Liang Song, Heye Zhang

**Affiliations:** 1 School of Biomedical EngineeringSun Yat-sen University 26469 Guangzhou510275China; 2 Insight Lifetech Company Ltd.Shenzhen518052China

**Keywords:** iFR, object point localization, SFRA, 1D SE block

## Abstract

Accurate iFR calculation can provide important clinical information for intracoronary functional assessment without administration of adenosine, which needs to locate object points in the pressure waveforms: peak, the dichrotic notch and the pressure nadir at the end of diastole. We propose a DFENet that is capable of locating object points to calculate iFR accurately. We first design a SFRA into DFENet with the idea of DenseNet. To avoid overfitting when dealing with sparse signals, we set appropriate number of network layers, growth rate of dense blocks and compression rate of transition blocks in 1D DenseNet. Then, we introduce a feature enhancement mechanism named 1D SE block for enhancing inconspicuous but vital features from SFRA, which guides DFENet to focus on these important features via feature recalibration. Finally, we prove an effective interaction mode between SFRA and 1D SE block to locate object points accurately. Adequate experiments demonstrate that DFENet reaches a high accuracy of 94.22%, error of 5.6 on object point localization of 1D pressure waveforms that include 1457 samples from 100 subjects via a cross-validation of Leave-One-Out. Comparison experiment demonstrates that the accuracy of DFENet exceeds other state-of-the-art methods by 3.35%, and ablation experiment demonstrates that the accuracy of SFRA and cSE exceed the other variations by 6.63% and 2.56% respectively. Importantly, we reveal how the DFENet enhance inconspicuous but vital feature by applying gradient-weighted class activation maps. DFENet can locate object points accurately, which is applicable to other signal processing tasks, especially in health sensing.

## Graphical abstract

In this paper, we develop a deep feature enhancement network (DFENet) for object points localization of 1D pressure waveforms. Experimental results show that the DFENet has obvious advantages in locating object points robustly, which means that the method can provide accurate instantaneous wave-free ratio (iFR) for clinical diagnoses and intracoronary functional assessment.

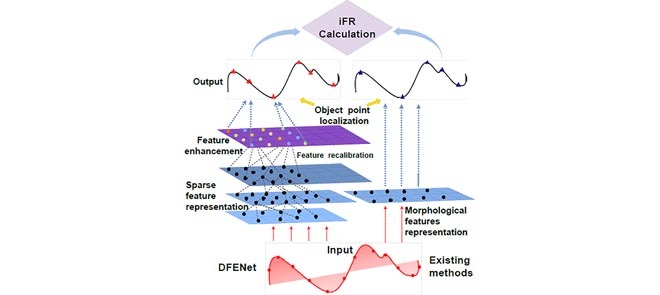

## I. Introduction

Accurate instantaneous wave-free ratio (iFR) calculation determined by one-dimensional (1D) aortic pressure waveforms has important clinical significance for intracoronary functional assessment. Clinically, there are two intracoronary physiologic indexes to diagnose coronary artery disease, which are fractional flow reserve (FFR) requiring the administration of adenosine and iFR without the administration of adenosine [1]–[4]. FFR is the most widely used index in clinical practice with a large body of evidence. However, iFR is gradually gaining more approvals from clinicians because iFR negates the need for administration of adenosine, saving time, reducing costs and side effects, and leading to improved adoption in the cardiac catheter laboratory [1]. Besides, studies have shown that iFR has diagnostic accuracy similar to that of FFR as independent measures of ischemia [5], [6]. iFR can be used as an indicator to diagnose the degree of coronary artery stenosis. Coronary artery lesions determined to have iFR ratios less than 0.89 and FFR ratios less than 0.8 currently are recommended to undergo further treatment with PCI [7], [8]. As it is still a newer technology, some providers consider an iFR ratio of 0.86 to 0.93 an area of uncertainty and recommend a hybrid approach utilizing evaluation with FFR. As a adenosine-free index of stenosis severity, the iFR is calculated by measuring the resting pressure gradient across a coronary lesion during the portion of diastole. The iFR is determined by a defined-time window that is decided over the wave-free period. The wave-free period is identified by accurately locating the object points on resting aortic pressure waveform. These object points include the peak, the dichrotic notch and the pressure nadir at the end of diastole. Moreover, it is widely accepted that the wave-free period is calculated the beginning 25% of the way into diastole and ending 5ms before the end of diastole, and the onset of diastole was determined from the dicrotic notch [1]. So iFR is calculated as the mean pressure distal to the stenosis during the diastolic wave-free period ($\bar {Pd}_{wave-free\,\,period} $) divided by the mean aortic pressure during the diastolic wave-free period ($\bar {Pa}_{wave-free\,\,period}$).

**FIGURE 1.**
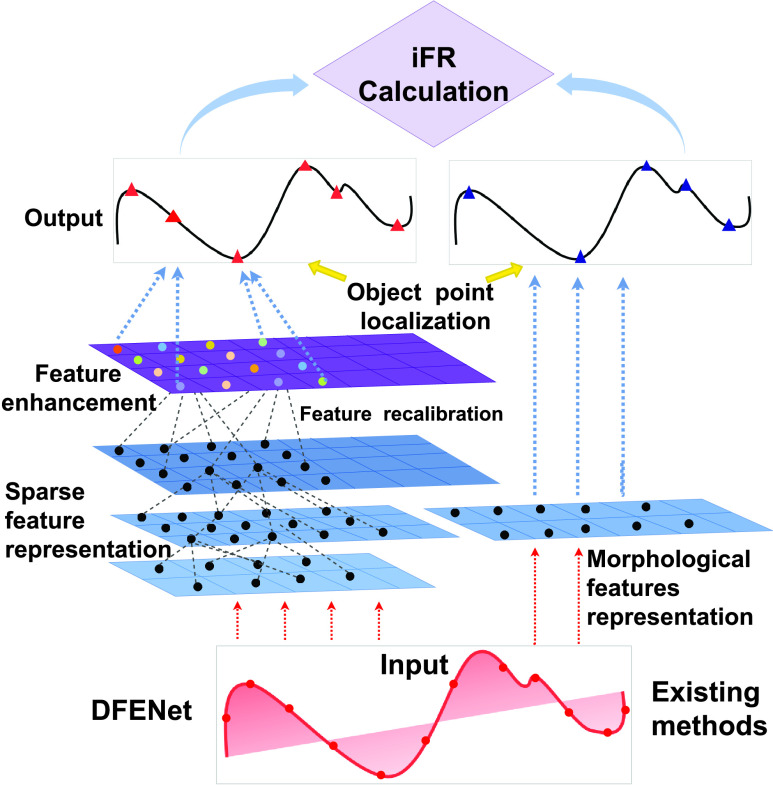
Examples of object point localization on 1D pressure waveform by DFENet. Compared with existing methods that only use low-level morphological features, DFENet is advantageous in locating object points comprehensively. The colorful small circles in feature enhancement step denote recalibrated feature with different weights.

The existing algorithms still have difficulty in locating object points robustly for calculation of iFR due to individual difference [9]. Fig. 1 shows that the features of 1D pressure waveforms are sparse morphologically. Besides, many object points on 1D pressure waveforms from subjects are inconspicuous intuitively. Clinically, a range of mathematical algorithms are proposed to analyze and locate object points of 1D pressure waveforms [1], [9]. They all focus on low-level morphological feature representation that are vulnerable for 1D pressure waveforms. Meanwhile, they are difficult to get the correlation between object points and other points that assists in locating inconspicuous object points. Therefore, the existing algorithms are difficult to obtain rich information included deep high-level feature for feature representation because of the sparsity of 1D pressure waveform. Moreover, they are hard to mine inconspicuous but vital features. So we need a powerful and robust approach to locate object points for the calculation of iFR. Obviously, deep learning is powerful to exploit high-level spatial information for outstanding feature representation automatically from the input data [10]–[12]. Besides, it is able to explore the correlation between object points and other points [13].

Recently, deep learning has become a powerful approach for solving various classification and regression problems of 1D signal. As early as the 1940s, Pitts and McCulloch *et al.* referred to the structure of biological neurons and proposed an abstract neuron model (MP) [14]. Hebb *et al.* put forward the theory of neuropsychology, which led to the birth of the first artificial neural network perceptron in the 1950s [15], [16]. Perceptron can learn and recognize some simple digital data. Multilayer perceptron has effect of nonlinear classification, which is very computationally intensive. In the 1980s, Hinton *et al.* proposed backpropagation (BP), which can solve complex nonlinear problems [17]. After that, a series of deep learning networks were developed. Typically, the deep belief network was developed by Hinton *et al.*, and its performance on the MNIST data set surpassed that of support vector machines (SVM) [18], [19]. In addition, typical deep learning architectures also include recurrent neural networks (RNN) [20], the stacked autoencoder (SAE) [21] and convolutional neural networks (CNNs) [22]. Deep learning has an outstanding feature learning on the 1D signals [23], [24]. In the field of 1D signals, deep learning methods are generally used to solve two classes of problems: 1) classification problems. 2) regression problems.

1) *Classification Problems:* Most attempts have adopted deep learning methods for classification problems of 1D signal, such as the classification of Electrocardiogram(ECG) [25]–[28], analysis of Electroencephalogram (EEG) [29], [30]. In [27], the authors proposed a 1D CNN that fused the feature extraction and classification into a single learning sample, which was valuable for a real-time implementation for heart monitoring and anomaly detection. The adaptive implementation of 1D CNN could extract handcrafted features and achieve the classification of long raw ECG data stream. In [31], the authors proposed a 1D-Convolutional LSTM neural network for EEG-based user identification system, which utilized the spatiotemporal features of the EEG signals with LSTM. In [29], the authors used a framework combined 1D CNN and SAE to classify EEG motor imagery signals. 1D CNN extracted the information of time, frequency and location on EEG. Then the deep network SAE was used to classify EEG records. In [32], the study introduced a dual-channel CNN that consisted of a 1D dense block and a two-dimensional (2D) dense block to extract the hierarchical spectral features and hierarchical spatial-related feature respectively for image classification. In [25], the authors proposed an eleven-layer deep CNN to automatically detect four different ECG class and obtain high accuracy.
2) *Regression Problems:* A few studies have focused on the regression problems of 1D signal. In [33], the study developed a LeNet-style ConvNet that had a six-layer deep CNN without dropout layers to detect and locate the waves in heartbeat, include the P-waves, the QRS-waves, and the T-waves.

Existing deep learning methods are hard to deal with object point localization task of 1D pressure waveform due to three unusual challenges. Firstly, it is a challenge to solve regression problems for 1D signal such as object point localization. The solution of object point localization demands highly concentrated feature learning. In [33], the authors proposed a six-layer CNN to locate the waves effectively. But the locations are not must at peak of the waves but just inside the wave intervals. It means that the proposed method is hard to locate exacted points and has a poor feature representation. Undoubtedly, the existing methods have been mostly concentrate on classification problems rather than regression problems for 1D signal. Secondly, the existing methods have difficulty to perform a feature representation for 1D sparse signal. The 1D pressure waveform with sparse features is different from the ECG, EEG or facial image that has a wealth of information generally. In [25], the proposed CNN was adopted to analyze ECG signals due to the complexity and non-linearity of them. Sufficient information from ECG could be utilized to automatically detect the different ECG segments. But it is hard to explore sparse features of 1D signal effectively. In addition, unlike facial feature point detection task, which is based on a large amount of available facial appearance information, the task of locating object point of 1D pressure waveform faces a sparse signal [34], [35]. It needs to build an appearance model for sparse signals and a model of the relationship between object points and points. Therefore, the existing methods to deal with the 1D pressure waveform are likely to have the problem of overfitting. Thirdly, the existing methods have difficulty to enhance inconspicuous but vital features of 1D signal. Fig. 1 shows that part of object points of 1D pressure waveform are inconspicuous but vital. The existing methods are mostly designed for feature learning and object classification, such as [27], [28]. There are no feature enhancement mechanism applied to 1D signal. [30] proposed a channel-wise competition mechanism to focus on vital and relevant EEG channels. But its function is just setting neurons to zero via channel-wise ranking, which is hard to realize enhancement of inconspicuous feature. Besides, the input data of network was changed into 2D picture to be trained. Therefore, the amount of parameters was increased.

To overcome the aforementioned challenges, we propose a deep feature enhancement network (DFENet) for 1D signal object localization task in this paper. As shown in Fig. 1, the proposed DFENet consists of a sparse feature representation architecture (SFRA) and a feature enhancement mechanism. The SFRA in the DFENet adopt the idea of 1D densely connected convolutional networks (DenseNet) to implement effective feature representation of 1D sparse signal [36]. SFRA is capable of densely connecting learned features to obtain rich information include high-level spatial features and low-level features, and also has powerful generalization ability. Because of the sparsity of 1D pressure waveforms, the main settings of 1D DenseNet are changed to ensure that there will be no overfitting problems. The feature enhancement mechanism of DFENet adopt the idea of Squeeze-and-Excitation (SE) block [37] to enhance inconspicuous but vital features of 1D signal, which is named 1D SE block. The 1D SE block is capable of enhancing the features by recalibrating features. Moreover, we find an effective interaction mode of SFRA and 1D SE block inside the DFENet for object point localization accurately by adjusting the position of them in DEFNet.

The primary contributions of this work are summarized as follows:

• We propose DFENet for object point localization on 1D sparse signal. We employ proposed DFENet to locate object points on 1457 samples of 1D pressure waveforms from 100 subjects. We achieve a high accuracy on object point localization for calculation of iFR.
• We design SFRA into DFENet for 1D signal. SFRA adopt the idea of 1D DenseNet, which set appropriate number of network layers, growth rate of dense blocks and compression rate of transition blocks to deal with sparse signals. It can obtain rich information and avoid overfitting for effective sparse feature representation.
• We introduce 1D SE block into DFENet for enhancing inconspicuous but vital features that come from SFRA. This mechanism is capable of guiding DFENet to focus on these important features via feature recalibration strategy.
• We prove an effective interaction mode between SFRA and 1D SE block by adjusting the position of SFRA and 1D SE block inside DFENet for object point localization accurately.

The rest of this paper is organized as follows: In Section II, we present the method of DFENet. In Section III, we provide adequate experimental results to demonstrate the effectiveness and performance of DFENet. In Section IV, we draw a conclusion on this paper.

## II. Methods

### A. An Overview of DFENet

**FIGURE 2.**
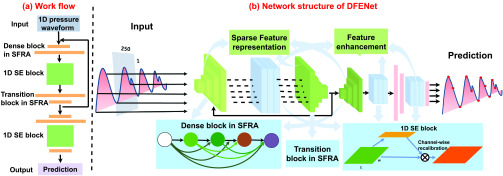
(a) Work flow of DFENet. The SFRA conduct feature connection, compression and prediction. The 1D SE block performs feature recalibration. (b) Network structure of DFENet. The input is 1D pressure waveforms and output is the location of the object points. SFRA captures effective features from sparse signals with the idea of feature dense connection. 1D SE block enhances inconspicuous but vital features from SFRA via feature recalibration.

**FIGURE 3.**
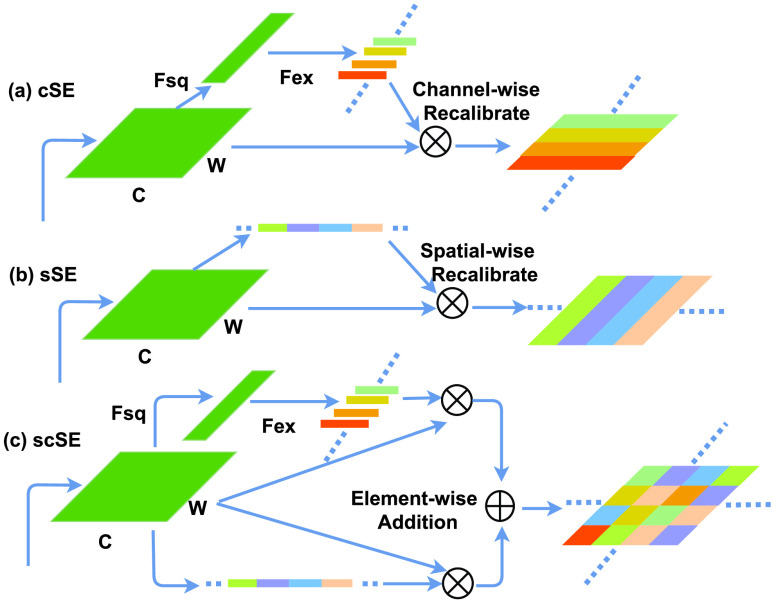
Three types of 1D SE block. The architecture of cSE, sSE and scSE are shown in (a), (b), and (c), respectively. $U$ denotes the combination of channels.

Our DFENet consists of a sparse feature representation architecture (SFRA) shown in Section II-B and a feature enhancement mechanism (1D SE block) shown in Section II-C. Meanwhile, an effective interaction mode of SFRA and 1D SE block inside DFENet is proved shown in Section II-D. Fig. 2 shows the details of our work flow and network structure. The inputs are the 250 points extracted from 1D pressure waveforms. And the output of DFENet are the localizations of object points. Firstly, the SFRA inside DFENet obtain a wealth of details from input data via dense connection operation, which ensures SFRA acquire an ability feature learning robustly. Meanwhile, it eliminates redundant information for sparse 1D pressure waveforms via compressing the depth and filters appropriately. Then, 1D SE block inside DFENet provides feature enhancement for enhancing inconspicuous but vital features that come from SFRA. It utilizes global pooling and a gating mechanism to recalibrate features as illustrated in Fig. 3 (a). Finally, we adjust the position of SFRA and 1D SE block inside DFENet to obtain an effective interaction mode. This interaction mode is capable of locating object points accurately.

### B. SFRA for Feature Representation

To obtain effective feature representation of 1D pressure waveform, we design SFRA for feature learning that employ the idea of densely feature connection. Considering powerful ability of DenseNet in extracting sufficient and effective information, we shrink the 2D DenseNet to develop SFRA for 1D signal. We compress the depth and number of 1D filters appropriately. It is thus able to eliminate redundant information for 1D signal. SFRA consists of three dense blocks to connect the features densely, two transition blocks without bottleneck layer to compress channels, and a prediction block to predict object points. Fig. 2(b) shows that SFRA inside DFENet is able to acquire a feature representation through dense block, transition block, and prediction block.

The densely feature connection of SFRA is attributed to the dense block. It ensures that the features learned in the previous layer can be directly connected to any layer of all subsequent layers. When the distance between the two ends of the network contracted, the gradient would backpropagate from the output to the input more easily [38]. Therefore, the dense block effectively alleviates the vanishing gradient problem. It is composed of four convolution blocks with multiple convolution filters, which connect together before going into next block. The convolution block is made up of three composite functions: a Batch Normalization (BN) [39], a rectified linear units (ReLU) [40], a convolution layer with a kernel of 3. The BN layer reduces internal covariate shift, which can accelerate network convergence speed and make training easier. The ReLU layer increases the nonlinear relationship between layers of the neural network and lightens overfitting. The dense block in SFRA is capable of collecting sufficient features to represent contextual information in the 1D pressure waveform. Hence, the $l_{th}$ layer has access to all previous feature maps $x_{0}, \cdots, x_{l-1}$, as input:

\begin{equation*}x_{l}= R_{l}([x_{0}, x_{1}, \cdots, x_{l-1}])\tag{1}\end{equation*}

where $[x_{0}, x_{1}, \cdots, x_{l-1}]$ means the concatenation of the feature maps in layers $0,\cdots, l-1$. The $R_{l}$ means the three composite functions of convolution block.

The transition block effectively compresses the original channels to decrease parameters and cuts down the feature maps to unified input, which also make sure the features go smoothly to the next block. It consists of a BN, a ReLU, a convolution layer with a kernel size of 1, an average pooling layer that could reduce feature dimension. The channels would be increased after the dense block that has the function of feature dense connection. Then the channels would be reduced after transition block that has the function of feature compression. Considering the unique sparsity and contextuality of the 1D pressure waveform, we conduct a couple of considerable settings. The number of dense blocks is set at 3. And the appropriate number of network layers, growth rate of dense blocks and compression rate of transition blocks are set in SFRA. To protect the valuable information, the dropout layer is not set in the network.

The prediction block applies ReLu activation function to predict the object points of 1D pressure waveforms, which is different from the image classification used softmax function.

### C. 1D SE Block for Feature Enhancement

The feature enhancement mechanism of DFENet is attributed to 1D SE block with a feature recalibration strategy for 1D signal. The 1D SE block employs a global pooling operation to capture contextual information and a lightweight gating mechanism to enhance representational power by modelling channel-wise relationships. Therefore, 1D SE block is capable of activating useful features and suppressing useless features. Because SFRA is difficult to characterize inconspicuous but vital feature in 1D pressure waveform, 1D SE block could make up for its shortcomings and strengthens the correlation between object points and other points for feature learning of inconspicuous object points. 1D SE block is already applied for image classification [41] and segmentation [42], which all yield an extraordinary performance. However, 1D SE block in this work is used on 1D signal for the first time. 1D SE block is a computational unit which can be built for any given transform $F_{tr} $: $X\rightarrow U$, $X\in \mathbb {R}^{W'\times {C}'}$, $U\in \mathbb {R}^{W\times {C}}$. It is committed to improving the sensitivity of the network to informative features so that subsequent transformations are able to take advantage of these features and suppress less useful features. Thus, explicitly modelling channel interdependencies is employed to achieve this function through two steps: squeeze operation ($F_{sq}$) that can turn each 1D signal’s feature channel into a collection of the local descriptors and excitation operation ($F_{ex}$) that can generate distinctive weights for each feature channel and reweight the original features. The SE block can recalibrate filter responses, which means that it is more likely to acquire critical features.

For the problem of exploiting channel dependencies from 1D pressure waveform, every learned filter operates with a global average pooling to catch contextual information outside of the local receptive field. The $F_{sq}$ of our 1D SE block can be represented as follow:

\begin{equation*}z_{k}=F_{sq}\left ({u_{k} }\right)=\frac {1}{W}\sum _{i=1}^{W}u_{k}\left ({i }\right)\tag{2}\end{equation*}

where $U=\left [{ u1,u2,\cdots,u_{c} }\right]$ can be explained as the collection of output feature maps, k is the number of filters, $z\in \mathbb {R}^{k}$ is produced by feature map U with length W, $z_{k}$ is $k^{th}$ element of z.

The $F_{ex}$ of our 1D SE block is aimed at fully capturing channel-wise dependencies. It utilizes a gating mechanism of sigmoid function:

\begin{equation*}s=F_{ex}(z,W)=\alpha _{2}(g(z,W))=\alpha _{2}(W_{2}\alpha _{1}(W_{1}z))\tag{3}\end{equation*}

where $\alpha 1$ is the sigmoid function, $\alpha _{2}$ is the RuLU function and parameters $W_{1}\in \mathbb {R}^{\frac {C}{r}\times C}$, $W_{2}\in \mathbb {R}^{C\times \frac {C}{r}}$, r is the reduction ratio that is set to 8. Two fully connected layers and a rectified linear activation are utilized to parameterize the mechanism.

At last, 1D SE block has a final output, which is obtained by the operation as follow:

\begin{equation*}\hat {X}_{c}=F_{scale}\left ({u_{c},s_{c}}\right)=s_{c}\cdot u_{c}\tag{4}\end{equation*}

where $\hat {X}=\left [{ \hat {X}_{1},\hat {X}_{2},\hat {X}\cdots,\hat {X}_{C}}\right]$, $F_{scale}\left ({u_{c},s_{c}}\right)$ refers to channel-wise multiplication between the feature map $u_{c}\in \mathbb {R}^{W}$ and scalar $s_{c}$, which takes the rescaled weight that weighted to the original feature to boost feature discriminability. The output $u$ with the activations is rescaled thought the channel-wise multiplication. To learn more useful information from the spatial level, 1D SE block is created with two variants [43]. Fig. 3 shows three structures included spatial squeeze and channel excitation block (cSE), channel squeeze and spatial excitation block (sSE), and concurrent spatial and channel squeeze & channel excitation block (scSE). The cSE block has already been introduced above, which was a clever structure that enhances useful feature. The sSE block applies a sigmoid layer $\alpha _{1}\left ({\cdot }\right)$ to rescale activations and a convolution layer to achieve a squeeze operation whose kernel is 1 and stride is 1. The input tensor is $U=[u^{1,1},u^{1,2},\cdots,u^{p,q},\cdots,u^{H,W}]$, where $p\in \left \{{ 1,2,\cdots,H }\right \}$ and $q\in \left \{{ 1,2,\cdots,W }\right \}$, $u^{p,q}\in \mathbb {R}^{1\times 1\times C}$ means spatial location $(p,q)$. And the operation of sSE block is below:

\begin{align*}U_{sSE}=\alpha _{1} (l_{1,1})u^{1,1},\cdots,\alpha _{1}(l_{p,q})u^{p,q},\cdots,\alpha _{1}(l_{H,W})u^{H,W} \\\tag{5}\end{align*}

$\alpha _{1}(l_{p,q})$ means the relative importance of an information $(p,q)$. $U_{sSE}$ squeezes the feature map along the channel and excites spatially, which could learn spatial information. Recalibration operation in $U_{sSE}$ has function of ignoring irrelevant spatial location and enhancing the relevant ones between points. The scSE block is a combination of the two SE blocks above, which can contain global spatial information and provide a global receptive field at each stage of the network. Its receptive field implements channel squeezing through the global average collection layer, which is different from sSE block. In addition, The channel squeeze operation of scSE is also performed by a convolution layer with a kernel of 1. In summary, scSE block pays more attention on assisting object point prediction. However, compared with the cSE block and sSE block, the parameters amount of scSE block is increased and the model may not be able to avoid overfitting when dealing with our sparse signals.

### D. Position of SFRA and 1D SE Block Inside DFENet for Object Point Localization

**FIGURE 4.**
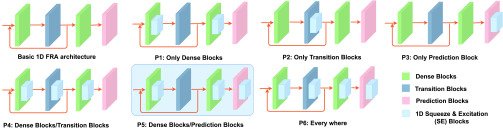
Combinations of 1D SE block and SFRA inside DFENet. The dense block, transition block and prediction block are inside SFRA. The recommended configuration of P5 is highlighted.

Effective interaction mode is attributed to appropriate position of SFRA and 1D SE block inside DFENet. Different position of SFRA and 1D SE block inside DFENet has different ways of information flow for the whole network, which create distinct collective effect. The collective effect includes feature representation and enhancement effect. Therefore, effective interaction mode of them is able to achieve prominent performance of DFENet. We explore six positions of SFRA and 1D SE block inside DFENet to find effective interaction mode of them shown in Fig. 4. The six possible configurations listed below:

P1: Position after dense block.
P2: Position after transition block.
P3: Position after prediction block.
P4: Position after dense block and transition block.
P5: Position after dense block and prediction block.
P6: Position after dense block, transition block and prediction block.

**FIGURE 5.**
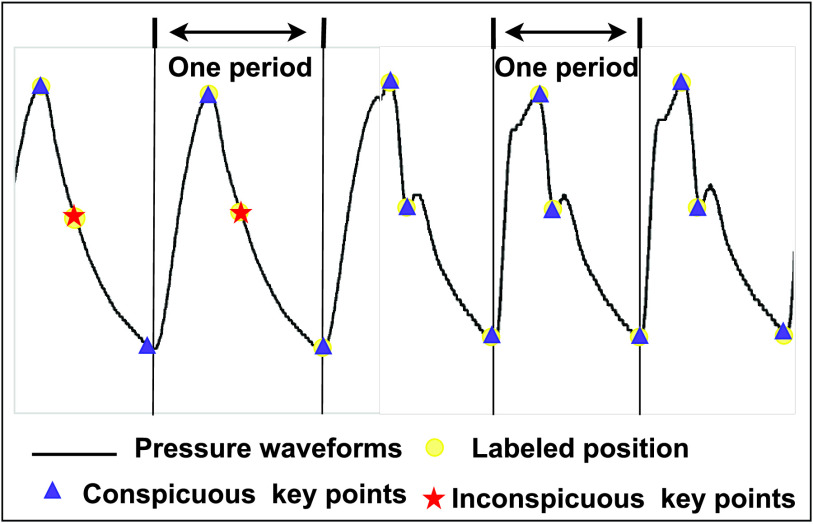
Qualitative results generated from the DFENet. The blue dots and red small star represent predicted points.

**FIGURE 6.**
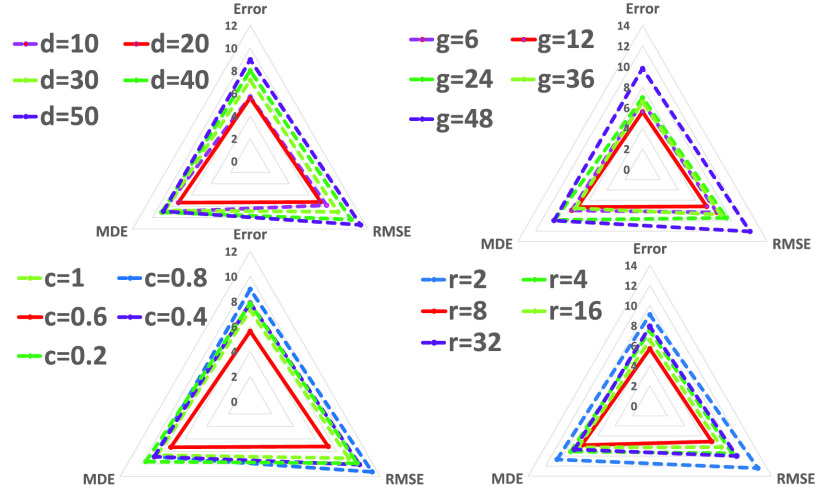
Comparison of effect with different values of basic initial parameters in DFENet.

**FIGURE 7.**
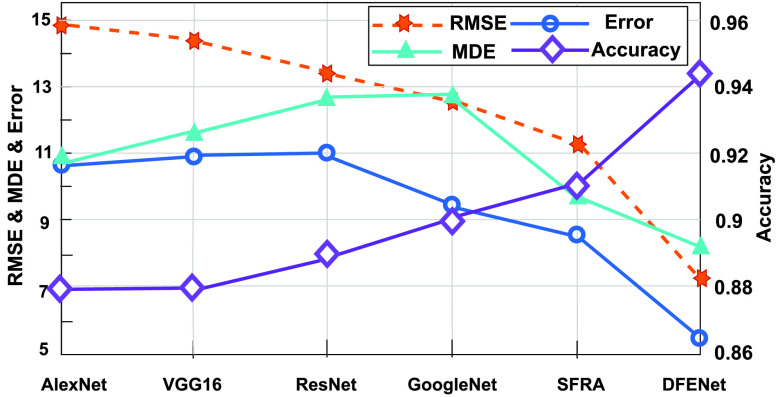
Comparison of RMSE, MDE, Error and accuracy between different methods. The DFENet has the lowest RMSE, Error and MDE (left axis). Besides it has the highest accuracy outperform five other methods (right axis). The ranges on both sides of the ordinate are 5~15 and 0.86~0.96, respectively.

**FIGURE 8.**
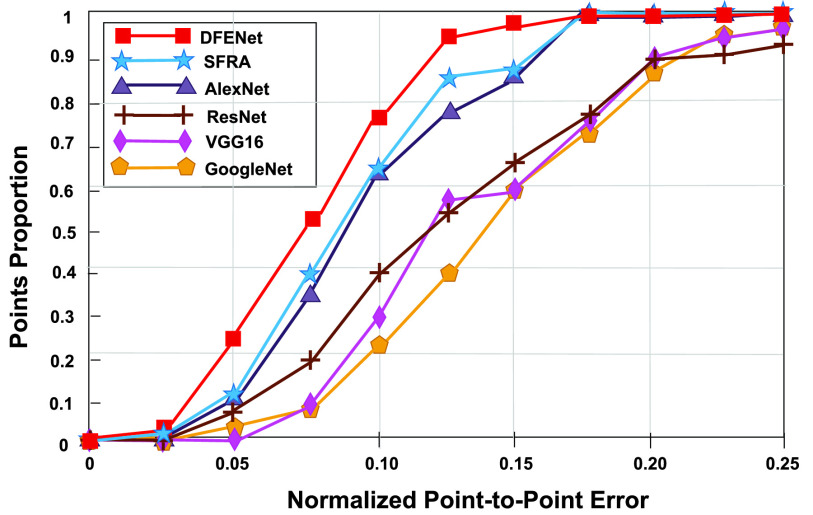
Comparison of cumulative error distribution (CED) curves on the test data. We set the abscissa $e_{i}$ in the range of $0\sim 0.25$ and the ordinate in the range of 0~1.0. The DFENet obtain a larger proportion in the same error $e_{i}$, indicating that it has the stronger display.

**FIGURE 9.**
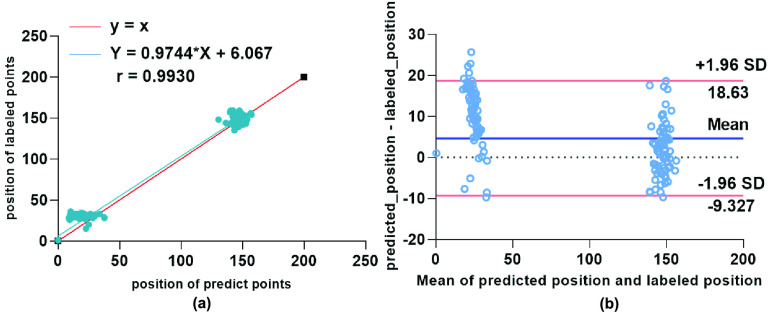
Correlation analysis (a) and BlandAltman analysis (b) shows the agreement between the predicted positions of object point determined by automatic localization and labeled positions.

**FIGURE 10.**
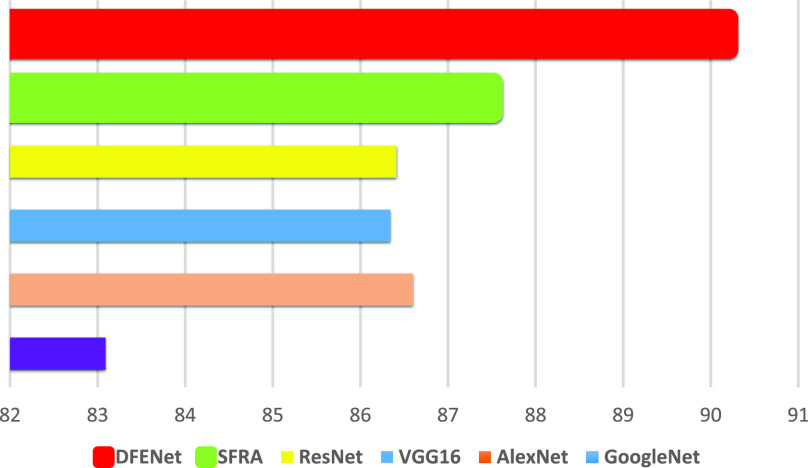
Comparison of accuracy of different methods on the test data from different hospital centers.

As can be seen in Fig. 4, multiple combinations have more blocks and feature connections than single combination in the network, which mean more parameters created in the network correspondingly. However, an overly complex network with excessive parameters might bring the vanishing gradient problem. 1D SE block has power of enhancing useful features and suppress useless features, which is beneficial to increase gradient flow and eliminate much redundant information. However, too much block participation may result in too many parameters.

## III. Result and Discussion

**FIGURE 11.**
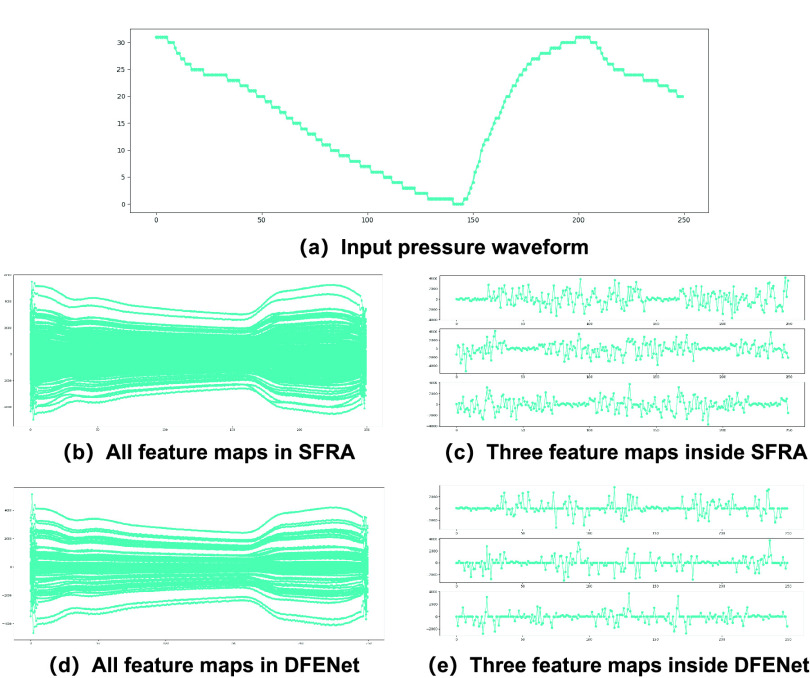
Feature maps in the same position of SFRA and DFENet. (a) shows original input data, (b) and (d) show all feature maps, (c) and (e) show three specific feature maps of DFENet and SFRA. Both (d) and (e) show that DFENet could suppress redundant information compare to SFRA.

**FIGURE 12.**
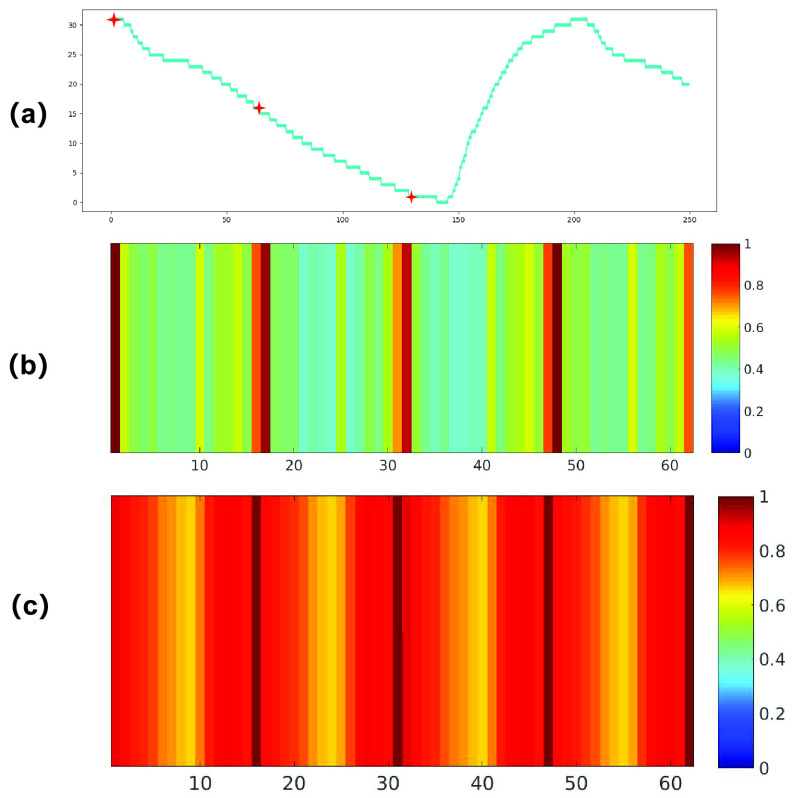
Grad-CAM visual explanation for locating object point by DFENet and SFRA. (a) Input pressure waveform consists of three labelled object points. (b) Grad-CAM explanations of the feature enhancement in DFENet. (c) Grad-CAM explanations of the feature enhancement in DFENet. The Grad-CAM visualization is calculated for the last convolutional outputs.

To verify the effectiveness and performance of DFENet, we run four types of experiments as follows. Firstly, in order to validate effective feature representation by SFRA and feature enhancement by 1D SE block, we compare the performance between different ablated variations of SFRA and ablated variations of 1D SE block by adopting performance metrics (see Section III-A). The experiment demonstrates that the accuracy of SFRA surpassed the other variations by 6.63% and the accuracy of cSE surpassed the other variations by 2.56%. Secondly, to verify the effectiveness of DFENet, we compared it with the state-of-the-art methods. DEFNet achieves higher accuracy that surpasses other state-of-the-art methods 3.35%. Thirdly, in order to observe the feature enhancement effect of 1D SE block inside DFENet, the method of gradient-weighted class activation maps (Grad-CAM) is applied to provide insight into how DFENet enhances inconspicuous but vital features (see Fig. 11 and Fig. 12). Finally, to verity effective interaction mode of SFRA and 1D SE block in DFENet, we compare the performance of DFENet and other five combinations.

### A. Data Acquisition and Experimental Settings

#### 1) Data Acquisition

In this study, we establish a dataset from Insight Lifetech Co. Ltd. A total of 1457 samples of 1D pressure waveform complexes from 100 unparalleled subjects (64 men and 36 women) are adopted. The 100 subjects come from two different hospitals. Because the number of samples collected by each person is not the same, we assign data sets by the number of people. The 100 subjects are assigned to training set (n = 75), validation set (n = 24) and test set (n = 1). The process consists of two steps. The first step is data pre-processing for the DFENet. The second step is the training and testing of the DFENet. The pre-processing produce is described as follows. The first step is extracting pressure waveform complexes from 1D pressure waveforms of every subject. The waveforms are periodic and every period is a complex, so we separate them firstly. Extracting complexes have different lengths because of individual differences. The networks need fixed-length signals to feed. Thus, the second step is making all complexes of waveforms the same length of 250 data point signals by padding the signals with the last value of every period. In addition, Labelling all data is completed by three experienced clinicians. The inter-observer error ranged between 0.43 and 5.11% (average: 1.61%).

#### 2) Experimental Settings

All the proposed deep learning networks are implemented using a Python tool in the Keras framework (Tensorflow as backend). The training is implemented on a Linux (Ubuntu 16.04) desktop computer with the Intel Xeon CPU E5-2620 and NVIDIA TITAN XP GPU with 24G memory. All pressure waveforms from the same subject are put into the same data set to test our model. Note that all the reported results are followed by a cross-validation of Leave-One-Out. The DFENet model is trained on training subset which is consist of waveforms of 1s long (sampled at 250Hz).

#### 3) Performance Metric

Reliability-based metric. The reliability-based metric measures the root mean squared error (RMSE), which certainly proves the performance of SFRA and 1D SE block. In the experiment, loss function needs to determine the distance between the predicted location of the key points and the marked annotation of the pressure waveforms. RMSE can indicate the quality of the match, going from the best match to the worst match, whose range is $\left [{ 0,\infty }\right] $. It is given by:

\begin{equation*}{RMSE=\sqrt {\frac {1}{k}\sum _{i=1}^{k}(m_{i}-n_{i})^{2}}}\tag{6}\end{equation*}

Here, k = 3, which means the three dimensions to the output. Every dimension represents the locations of predicted object point. $M_{i}$ is the $i_{th}$ predicted location and $n_{i}$ is the $i_{th} $ actual object point locations that experienced clinicians marked. As a loss function, RMSE is used into the neural network to train the weights with the back-propagation method [44]. The accuracy of prediction can be obtained by computing RMSE [33].

Accuracy metric. In order to more intuitively see the effect of the entire networks, we design an evaluation metric based on RMSE. Considering there are 250 points, if the prediction is randomly determined, then the average accuracy would be 125 data points. Therefore, accuracy can be acquired from Eq. (7):

\begin{equation*}{Accuracy=\frac {125-RMSE }{125}}\tag{7}\end{equation*}

The accuracy was measured on test data of the entire subject.

Average error metric. The average error is a common evaluation indicator for the performance test, which indicates the distance between predicted point and labeled data in the same cross-validation test. In the field of face recognition, the average error is widely used to evaluate method performance. It is defined as follow:

\begin{equation*}{Error=\frac {1}{M}\sum _{i=1}^{M}\frac {1}{N}\sum _{j=1}^{N}\sqrt {(\hat {x}_{i, j}-x_{i, j})^{2}}}\tag{8}\end{equation*}

where $\hat {x}_{i,j}$ and $x_{i,j}$ denotes predicted localization and the ground truth, respectively, $M$ is the number of samples, $N$ denotes the number of landmarks on one piece of test data.

*Mean Distance Error (MDE):* To see the distance error of two point on pressure waveform, we design the $MDE$ which is a measurement index for mean error of predicted point-to-point. It is defined as follow:

\begin{equation*}{MDE=\frac {1}{M}\sum _{i=1}^{M}\frac {1}{H}\sum _{j=1}^{H}\left |{(\hat {x}_{j+1}-\hat {x}_{j})-(x_{j+1}-x_{j}) }\right |}\tag{9}\end{equation*}

where $\hat {x}_{j+1}$ and $\hat {x}_{j}$ is the $(j+1)_{th}$ and $j_{th}$ predicted localization on pressure waveform, ${x}_{j+1}$ and ${x}_{j}$ is the $(j+1)_{th}$ and $j_{th}$ labeled localization, $M$ is the number of samples, H is the number of segments of distance between points in each sample and $H=2$ here.

Cumulative error distribution (CED). It is used to the cumulative error of all points that can take a comprehensive comparison for DFENet. The abscissa indicates the normalized point-to-point error defined as follow:

\begin{equation*}{e_{i}=\frac {\left \|{ x_{i}-x_{i}^{*} }\right \|_{2}}{d_{MD}}}\tag{10}\end{equation*}

where $x_{i}$ means the predicted value of location, $x_{i}^{*}$ means the ground truth and $d_{MD}=125$ in our experiment. The ordinate indicates the proportion of the sample smaller than the normalized error to the total sample.

#### 4) Parameter Initialization

In order to set the appropriate initial parameter value of DFENet to show its best effect, we run the parameter initialization experiment. Basic initial parameters include the number of network layers in DFENet, the growth rate of dense block, the compression factor of transition block and the reduction ratio of 1D SE block. Firstly, different number of network layers $d$ in DFENet means different memory cost of whole network. As $d$ increases, the memory cost increase and the ability of the prediction would be affected. Fig. 6 (a) shows performance of DFENet with different depths of 10, 20, 30, 40, and 50, which denotes that DFENet display the best ability when $d=20$. Secondly, growth rate $g$ of dense blocks means $g$ feature maps generated by function $R_{l}$ in $l_{th}$ layer. Fig. 6 (b) shows that small growth rate ($g=6, 12$) is sufficient to acquire efficient results on the test set. One explanation for this is that each layer could connect all the preceding feature maps in its block and thus has access to global information. DFENet would have a large amount of parameters when $g$ is large, which would cause overfitting. Thirdly, compression factor $c$ of transition block denotes that reducing $(1-c)m$ ($m$ is feature maps from dense block) feature maps in transition block to improve model compactness. Fig. 6 (c) demonstrates that DFENet has the best performance when $c=0.6$. Finally, reduction ratio $r$ of 1D SE block denotes the dimensions of output feature channels would be $\frac {1}{r}$ of the input. It varise the capacity and computational cost of the 1D SE blocks in our model. To investigate effect of this hyperparameter, we conduct experiments with DFENet for a range of different $r$ values ($r = 2$, 4, 8, 16, 32). Fig. 6 (d) demonstrates DFENet perform best with $r$ value of 8. In addition, our batch size is set to 32. An Adam optimizer is applied to the training procedure. Because of the sparsity of 1D pressure signal, the dropout layer is not set. The training epoch is set as 100 and the initial learning rate was 0.0001.

### B. Ablation and Comparison Experiment

In order to illustrate the power of effective feature representation of SFRA and feature enhancement of cSE, we conduct the ablation experiments that compared them with related variants. The details of the experiment are displayed below, which indicate that the effectiveness of DFENet. After that, we perform the comparison experiment between DFENet and five other methods to reveal the effectiveness of DFENet. Then, we apply correlation analysis and Bland-Altman analysis to evaluate our method. Finally, we explore the practical application effect of DFENet.

We investigate four SFRA variants and two SE variants, which are utilized to test our data compared with our DFENet. Due to our SFRA adopt the idea of DenseNet, we adopt different configurations of 1D DenseNet, which are respectively 1D DenseNet-121, 1D DenseNet-169, 1D DenseNet-201, 1D DenseNet-161. Meanwhile, two SE variants displayed in the method are used to compare the performance. The specific evaluation metrics include accuracy, error, and RMSE are utilized to compare the performance of various methods in the experiments. Compared to [45], our evaluation metric is more comprehensive and the performance is very impressive. Table 1 shows the overall results of ablation experiment. The accuracy of the DFENet is 94.23%, which demonstrates the superiority of SFRA and 1D cSE block.

**TABLE 1:** Overall Results of Ablation Experiment With Three Metrics. The Highlighted One Represent the Best Performance Compared With Four SFRA Ablation and Two SE Variants

	DenseNet-121+cSE	DenseNet-169+cSE	DenseNet-201+cSE	DenseNet-161+cSE	SFRA+sSE	SFRA+scSE	SFRA+cSE (DFENet)
RMSE	15.32	18.22	18.16	16.1	10.41	15.4	9.5
Accuracy(%)	87.6	85.4	85.5	87.1	91.67	87.67	94.23
Error	12.32	13.75	13.88	12.2	8.02	11.97	5.6

Meanwhile, we compare the performance of DFENet and five state-of-the-art methods included 1D AlexNet, 1D VGG16, 1D GoogleNet, 1D ResNet, SFRA. As we can see, the performance of DFENet (accuracy of 94.23%, error of 5.6) prevails the 1D AlexNet (88.26%, 10.89), 1D VGG16 (88.1%, 10.72), 1D GoogleNet (89.98%, 9.69), 1D ResNet (89.13%, 10.24), and SFRA (90.88%, 8.49). Fig. 7 shows that DFENet has the lowest RMSE of 7.21, error of 5.6 and MDE of 8.33 in a Leave-One-Out test. Meanwhile, Fig. 7 shows that DFENet yields high accuracy rate (94.23%) between the predicted point and labeled data in the same cross-validation test, which proves powerful ability of DFENet. We can also see that SFRA has better ability to characterize sparse signals compared with others. Fig. 8 shows DFENet has the better error distribution than the others on the test data, whose curve is on the left. Referring to the above figures, we can see that previous approaches include 1D AlexNet, 1D VGG16, 1D GoogleNet, and 1D ResNet are hard to get the accurate localization of all object points robustly. This manifests that it take advantage of extracted features more robust compared against other approaches. All results below all prove that the DFENet can determine accurate localization of object points to calculate iFR, which is represented visually in Fig. 5.

Then, correlation analysis and Bland-Altman analysis are applied to evaluate the consistency between DFENet and the manual drawing method. Correlation analysis is utilized to explore the correlation of both predicted result and labeled position. Bland-Altman analysis is applied to assess the agreement between DFENet and the manual drawing method. Fig. 9 shows that DFENet achieves high correlation (The correlation coefficient $r=0.9930$) on locating object points when compared with experts. The mean difference between automatic and manual measurements of locating object point is 4.653. Yet the red lines in Fig. 9 (b) represent the 95% limits of agreement. 97.7% of the bias points are within the confidence intervals. These result plots illustrate that our DFENet has a high agreement with the referenced object points from experienced experts, which reveals the clinical potential of DFENet.

Finally, to prove that DFENet is suitable for real application scenarios, We use the data of one hospital as the training set (74 subjects) and the data of the other hospital as the test set (26 subjects) to test our model. Fig. 10 shows that DFENet has the best performance among all methods, which denotes its effect on actual scenarios.

### C. Feature Enhancement of 1D SE Block

To verity the feature enhancement of 1D SE block, we conduct two experiments on the same test data. Firstly, to understand how 1D SE block enhances the features learned from SFRA, we compare the feature maps of DFENet and SFRA. Secondly, we apply gradient-weighted class activation maps (Grad-CAM) to visualize learned features for enhanced effect display.

#### 1) Comparison of Feature Maps in DFENet and SFRA

To comprehend the feature enhancement of 1D SE block, we now try to analyze the function of feature enhancement for 1D pressure waveform during training. Note that the enhanced feature maps, similar to channel activation maps are element-wise multiplied with original feature maps from the SFRA. We could know the effect of enhanced mechanism via observing the feature maps. Firstly, in order to view the feature maps of all channels from one piece of test data, we run the experiment that extracts feature maps of all channels in the same position of SFRA and DFENet. The input data is shown in Fig. 11 (a), and the visualization result is shown in Fig. 11 (b) and Fig. 11 (d), which indicates that the 1D SE block reduces the redundant information distinctly. Then, we extract the feature map of three channels shown in Fig. 11 (c) and Fig. 11 (e). We can clearly see that many values of feature element come from SFRA are set to 0, which is likely considered as useless part. This indicates that 1D SE block has the function of feature weight re-adjustment.

#### 2) Grad-CAM Visual Explanation for Feature Enhancement

We are committed to visualizing focused region of network to explain feature enhancement. We apply Grad-CAM to DFENet and SFRA using a piece of test data. Grad-CAM is a visualization method which utilizes gradients to calculate the importance of the spatial locations in convolutional layers. Owning to the gradient is calculated with respect to object points, Grad-CAM result clearly show the location of interest. By observing the location that network focuses on, we attempt to look at how 1D SE block in DFENet enhancing vital features. Grad-CAM results could demonstrate the attended regions clearly shown in Fig. 12. We can clearly see that DFENet take more attention on vital feature than SFRA. That is, the proposed DFENet learns well to exploit information in target regions and enhance features from them.

### D. Comparison of Performance With Different Interactive Modes

To prove the effectiveness of interaction mode with 1D SE block and SFRA in DEFNet, we compare the performance of DFENet and other five possible configurations mentioned in Section II. The indicators for comparison of six combinations are nearly the same as above.

Table 2 shows that DFENet with configuration of P5 is more prominent than others. We apply four evaluation indices to compare the effect that include running time, parameters, average error and accuracy. In Table 2, several worth exploring behaviors about combinations are displayed. Firstly, we observe that 1D SE block leads to a clear promotion of localization accuracy and an obvious reduction of error at every position (P1-P6) of the network. Secondly, the combination of multiple blocks has more advantages than inserted 1D SE block of a single position, but there is little difference between them with respect to running time and parameters. As can be seen in Table 2, the performance of network which combined multiple blocks is better, such as P4, P5 and P6. Thirdly, the effect is more outstanding in the prediction block (P3), compare to dense block (P1) and transition block (P2) because of different interactive modes. Finally, the effect of P5 is the best among all combinations of multiple block, though they have less running time and fewer parameters than P6. In Tabel 2, we can observe that the parameters increased but the average running time of position did not increase much (249.7s at P1, 250.6s at P2, 256s at P4, 249.8s at P5). This shows that the computing power of the network is still strong after adding multiple SE blocks.

**TABLE 2:** Comparison of DFENet Performance in Different Configurations With the Depth of 20. $\text{P}1\sim$ P6 Denote Six Positions of 1D SE Block in the Network

Position	Running Time (s)	Parameters	Average Error	Accuracy (%)
No SE block	261.3	115,361	11.23	87.09
P1	249.7	119,007	10.15	89.25
P2	250.6	117,601	10.06	89.23
P3	238.4	119,213	6.75	93.09
P4	253	124,286	5.74	94.15
P5	249.8	125,898	5.6	94.23
P6	257.2	128,138	8.92	90.86

## IV. Conclusion

In this paper, we develop a deep feature enhancement network (DFENet) for object points localization of 1D pressure waveforms. The DFENet incorporates the feature representation architecture and feature enhancement mechanism named 1D SE block, obtaining an effective feature representation and enhancing inconspicuous but vital features. Moreover, we prove an effective interaction mode between feature representation architecture and 1D SE block. It overcomes the problem of non-robust localization of object points on 1D pressure waveforms by existing algorithms. Experimental results show that the DFENet has obvious advantages in locating object points robustly, which means that the method can provide accurate iFR for clinic diagnoses and intracoronary functional assessment.
